# Clinical and biochemical outcomes of cinacalcet treatment of familial hypocalciuric hypercalcemia: a case series

**DOI:** 10.1186/1752-1947-5-564

**Published:** 2011-12-05

**Authors:** Anne Qvist Rasmussen, Niklas Rye Jørgensen, Peter Schwarz

**Affiliations:** 1 Research Centre of Ageing and Osteoporosis, Department of Medicine, Glostrup University Hospital, Glostrup, Denmark; 2 Department of Clinical Biochemistry, Glostrup University Hospital, Glostrup, Denmark; 3 Faculty of Health Science, University of Copenhagen, Denmark

## Abstract

**Introduction:**

Familial hypocalciuric hypercalcemia is a rare benign autosomal-dominant genetic disease with high penetrance. In most cases, patients with familial hypocalciuric hypercalcemia experience unspecific physical discomfort or asymptomatic disease. These patients are typically characterized by mild to moderately increased blood ionized calcium and a normal to slightly elevated serum parathyroid hormone.

**Case presentation:**

Four female patients with familial hypocalciuric hypercalcemia with inactivating mutations in the *CaSR *gene were included in the treatment study. Three patients were related: two were siblings and one was the daughter of one of these. The ages of the related patients were 51 years, 57 years and 35 years. All three patients were carriers of the same mutation. The fourth patient, unrelated to the others, was 53 years old, and a carrier of a novel and previously unknown mutation leading to familial hypocalciuric hypercalcemia. All four patients were Caucasians of Danish nationality. Biochemically, all patients had elevated blood ionized calcium, serum parathyroid hormone, serum magnesium and total serum calcium, except one, whose serum parathyroid hormone was within the normal range prior to treatment. All patients were treated with cinacalcet in a dosage of 30 mg to 60 mg per day.

**Conclusion:**

Three months after the initiation of cinacalcet treatment, all our patients experiencing clinical signs of hypercalcemia had improved in self -reported well-being and in biochemical parameters. None of our patients suffered adverse events to cinacalcet treatment. Biochemical markers of calcium homeostasis were improved and remained stable during the observation period of 12 months (two patients), 24 and 36 months, in both the symptomatic and the asymptomatic patients.

## Introduction

Familial hypocalciuric hypercalcemia (FHH) is a rare, benign syndrome affecting the regulation of calcium metabolism. FHH is an autosomal-dominant genetic disease with high penetrance, caused by an inactivating mutation in the gene encoding the calcium sensing receptor, *CaSR*. The loss-of-function leads to decreased sensitivity of the CaSR to ionized calcium (Ca^++^), shifting the set-point for Ca^++^-regulated parathyroid hormone (PTH) release to the right [1]. This set-point shift is followed by an increased circulating level of PTH and subsequent hypercalcemia. However, blood (B)-Ca^++ ^is usually only moderately elevated in FHH patients, as is the level of serum (S)-PTH. In FHH, urinary calcium excretion is reduced and the renal tubular reabsorption of Ca^++ ^and ionized magnesium (Mg^++^) is increased. Patients with FHH usually present with S-Mg^++ ^concentrations in the upper end of the normal reference interval or it is only mildly elevated. In most cases, they do not experience as severe symptoms of hypercalcemia as those seen in primary hyperparathyroidism: cognitive dysfunction, kidney stones and skeletal complications. In most cases, patients with FHH are asymptomatic, or have a history of only mild symptoms, described as vertigo, uneasiness, feeling faint, tartar, muscle soreness or poor memory [2].

Calcimimetics are allosteric modulators of the CaSR. They increase the sensitivity and the expression of the CaSR, thus enhancing the CaSR signal transduction. Cinacalcet induces a transient left-shift of the calcium set-point by increasing the sensitivity of the receptor to Ca^++^, thereby decreasing the level of S-PTH [3,4]. For several years, the calcimimetic cinacalcet (Sensipar or Mimpara) has been used for the treatment of primary hyperparathyroidism (PHPT), hypercalcemia associated with parathyroid adenomas [5,6], parathyroid hyperplasia due to kidney disease [7] and parathyroid cancer [8]. It has been shown that, after one year of treatment, the average pre-dose reduction in S-PTH is 7.6% in PHPT patients [5]. In a five-year PHPT treatment study, cinacalcet treatment maintained a reduced S-calcium and S-PTH in the patients [9]. The effect of cinacalcet on S-calcium and S-PTH has also been shown to persist for at least three years of treatment without dose modifications in patients with secondary hyperparathyroidism (SHPT) [7]. However, no significant effect has yet been seen on bone mineral density (BMD) after long-term treatment of PHPT or SHPT patients with cinacalcet [5,10].

Cinacalcet is potentially a useful treatment of patients with intractable hypercalcemia caused by mutations in the *CaSR *gene [4,5]. In three patients with FHH with the amino acid changes *F809L *[11], *R220W *[12] and *R220Q *[13], and in one patient with neonatal hyperparathyroidism with the mutation *R185Q *[14], a significant reduction of hypercalcemia was observed during cinacalcet treatment.

In the present study, the clinical and biochemical effects of cinacalcet treatment from 12 months to 36 months are assessed in four female patients with FHH.

## Case presentation

Four female patients with FHH were referred to our outpatient clinic at the Research Centre of Ageing and Osteoporosis. Three of the patients carry the previously reported inactivating mutation (*R220W*) in the *CaSR *gene [15].

### Case 1

Case 1 (I-1) was a 51-year-old Caucasian woman of Danish nationality. She presented with elevated B-Ca^++ ^and S-calcium _(total), _together with slightly elevated S-PTH. Her calcium/creatinine clearance ratio was < 0.01 and she was a carrier of the inactivating (*R220W*) mutation in the *CaSR *gene. She experienced muscle cramps and muscle aches and poor memory. Furthermore, she suffered from paresthesia in her extremities, uneasiness and osteoporosis of the spine, with a BMD T-score (mean of lumbar vertebrae, L1-L4) of -3.1 prior to cinacalcet treatment.

### Case 2

Case 2 (I-2) was a 57-year-old Caucasian woman of Danish nationality. She presented with elevated B-Ca^++ ^and S-calcium _(total), _together with slightly elevated S-PTH. Her calcium/creatinine clearance ratio was < 0.01. This patient was a carrier of the inactivating (*R220W*) mutation in the *CaSR *gene and was the sister of patient I-1. She also experienced muscle cramps and muscle aches and poor memory prior to cinacalcet treatment.

### Case 3

**Figure 1:**
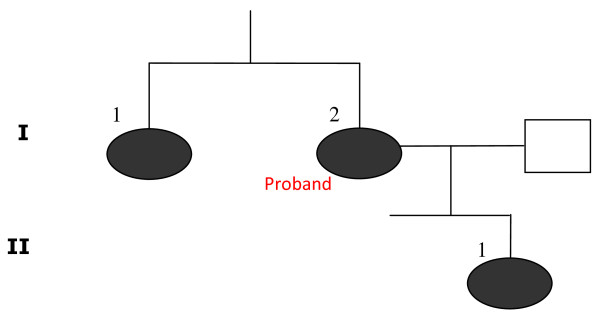
**Pedigree of the three related individuals diagnosed with FHH and carrying the *R220W *mutation**. Ι-1 and Ι-2 are symptomatic and have osteoporosis. ΙΙ-1 is asymptomatic with normal BMD. The amino acid substitution was found in the affected proband in Ι-2 and the same mutation was revealed in Ι-1 and ΙΙ-1. The filled symbol illustrates the individuals carrying the *R220W *mutation.

Case 3 (II-1) was a 35-year-old Caucasian woman of Danish nationality, and the daughter of Case 2 (see the pedigree in Figure 1). She presented with elevated B-Ca^++ ^and S-calcium _(total), _normal values of S-PTH. Her calcium/creatinine clearance ratio was < 0.01. This patient was a carrier of the inactivating (*R220W*) mutation in the *CaSR *gene. She was asymptomatic with normal BMD and no cognitive symptoms or other clinical signs of hypercalcemia.

All three related patients initiated cinacalcet treatment in a dosage of 30 mg once daily. After one to three months of treatment, all of them had their dose increased to 30 mg twice daily. Due to adverse events, such as eye palpitation, the dose for patient I-1 was reduced to 30 mg once daily. The eye palpitation disappeared within days.

The biochemical data are presented in Table 1. In all cases, their B-Ca^++ ^was found to be significantly lowered (range of reduction of 11% to 13%, *P *< 0.01) at first measurement (data not shown). The significant B-Ca^++ ^lowering persisted after one year (*P *< 0.01) and, in patient I-2, after three years (*P *< 0.01). In all three cases, their S-PTH levels were significantly decreased after one year of treatment (*P *< 0.05) and, in case I-2, after three years (*P *< 0.05) (Table 1). In patient I-1 and I-2, spine and hip BMD were re-evaluated after one year and three years of treatment. No significant improvement could be detected upon treatment.

**Table 1:** Biochemical data of the four patients treated with cinacalcet.

Biochemical data (normal range)	I-1		I-2				II-1			Case 4		
	baseline	1 year	baseline	1 year	2 years	3 years	baseline	2 months	1 year	baseline	1 year	2 years
S-Ca_(total) _(mmol/L) 2.20 to 2.60	**3.01**	**2.74**	**3.05**	2.58	**2.71**	**2.71**	**3.02**	2.51	NA	**3.13**	**2.86**	**2.87**
B-Ca++ (mmol/L) 1.18 to 1.32	**1.61**	**1.43**	**1.59**	**1.46**	**1.44**	**1.43**	**1.54**	**1.36**	1.26	**1.81**	NA	**1.47**
S-PTH (pmol/L) 1.6 to 6.9	**7.3**	**7.4**	**7.1**	4.4	3.3	1.9	3.5	6.2	2.9	**9.0**	**8.7**	**8.3**
S-phosphate (mmol/L) 0.76 to 1.41	**0.70**	0.84	0.83	0.93	1.00	NA	0.76	0.98	NA	0.96	0.93	0.79
S-Mg++ (mmol/L) 0.71 to 94	**0.96**	0.94	**1.12**	**1.00**	**0.97**	**0.97**	**0.97**	**0.95**	NA	**0.99**	0.94	0.94
S-creatinine (μmol/L) 50 to 90	55	55	59	52	57	NA	**49**	NA	NA	65	63	60
S-alkaline phosphatase (U/L) 35 to 105	**124**	**142**	89	105	94	NA	67	NA	NA	76	71	70
S-25(OH)-vitamin D (nmol/L) 50 to 178	38	NA	NA	NA	NA	NA	85	NA	NA	**31**	NA	NA
**BMD**												
T-score spine (L1-L4)	**-3.6**	**-3.7**	**-3.1**	**-3.0**	**-3.1**	**-3.0**	-0.1	NA	NA	-0.4	NA	-0.7
T-score hip	**-3.0**	**-3.0**	**-2.2**	**-2.0**	**-2.0**	**-2.0**	1.5	NA	NA	0.3	NA	0.0

I-1, I-2 and II-1 have an *R > W *substitution in codon 220 and Case 4 has a *G > R *substitution in codon 613. Data in bold are outside of the reference range. NA: not analyzed.

Prior to treatment, patient I-1 and patient I-2 suffered hypercalcemic symptoms. Both patients reported improved well-being after only a few months of cinacalcet treatment. They also no longer reported muscle cramps or muscle ache. However, the degree of reduced memory was unchanged. Patient I-1, who suffered paresthesia in her extremities and uneasiness, reported that her symptoms had disappeared upon treatment.

### Case 4

Case 4 was a 53-year-old Caucasian woman of Danish nationality. She presented with elevated B-Ca^++ ^and S-calcium _(total), _together with elevated S-PTH. Her calcium/creatinine clearance ratio was < 0.01. Patient was carrier of a novel heterozygous *de novo *inactivating mutation in the *CaSR *gene leading to FHH. This mutation was a missense mutation caused by a substitution of amino acid glycine (G) for arginine (R) in the transmembrane codon 613. This mutation has not yet been published. Patient 4 suffered clinical symptoms of tartar, vertigo and constipation. Measurement of her BMD showed values within normal range.

Patient 4 was initially treated with 30 mg cinacalcet once daily but the dosage was increased to 30 mg cinacalcet twice daily from month three.

The biochemical measurements are presented in Table 1. Her B-Ca^++ ^was significantly lowered by 11% (*P *< 0.01) after one month of treatment (data not shown). This significant B-Ca^++ ^decrease was maintained at a level even lower after increased the dosage and her B-Ca^++ ^was 19% below baseline (*P *< 0.01) after the second year (Table 1). Our patient's S-PTH was also significantly reduced (*P *< 0.05) (Table 1). After two years of cinacalcet treatment, her BMD was unaltered as compared to baseline.

Patient 4 also reported improved well-being after only a few months of cinacalcet treatment, with the disappearance of symptoms of tartar, vertigo and constipation. In addition, she reported no adverse events.

## Discussion

In the present series of cases, we observed significant improvement in the calcium homeostasis as evaluated by measurements of B-Ca^++^, S-calcium _(total), _S-magnesium and S-PTH, which is in accordance with other case reports [11-13]. Furthermore, the observations are in line with observations from PHPT patients with a cinacalcet treatment of up to five years duration [5]. In our case series, after observing for one year in two patients, two years in one patient and three years in the fourth patient, cinacalcet treatment was well tolerated without significant side effects. However, in one case, eye palpitation was reported at a dosage of 30 mg twice daily; this symptom disappeared immediately after dosage reduction. This observation is in accordance with previous studies published on PHPT [5,6]. In the three patients experiencing symptoms of hypercalcemia prior to cinacalcet treatment, we observed self-reported improvement. For the patient who did not report any hypercalcemic symptoms prior to treatment, no change in well-being was reported.

Thus, our biochemical data confirms the observations of Timmers *et al*. [11], Festen-Spanjer *et al*. [12], Alon and Vandevoorde [13] and Reh *et al*. [14], who all reported an effect of cinacalcet on biochemical parameters during short-term observation. Our long-term data show that the biochemical improvement towards normalization of B-Ca^++ ^and S-PTH persists for at least 36 months without side effects or the need for a change in dosage, as observed in one patient case.

In contrast, BMD did not improve in our observed patients treated with cinacalcet. However, the short observation time in two patients and only two and three years of observation in the remaining two patients may account for this. Although BMD data are sparse, our BMD data are in accordance with the observations of BMD reported on cinacalcet treatment in PHPT [5,10].

Our study does have limitations. The data presented are based on only four FHH cases of an open-label uncontrolled treatment and changes of hypercalcemic symptoms are based on self-reported life quality. However, our four cases are all clinically and biochemically very well described [15]. They have been followed in our outpatient clinic for more than 15 years.

## Conclusion

In conclusion, in four females with FHH we observed improvement in their B-Ca^++ ^and S-PTH upon cinacalcet treatment, without significant side effects. The improvement of the calcium homeostasis persisted for up to three years without the need for dose escalation. However, BMD does not seem to be improved during treatment. In addition, self-reported symptoms of hypercalcemia in three symptomatic patients improved or even disappeared whereas one asymptomatic patient did not report any changes in wellbeing. However, in order to fully document the usefulness of cinacalcet treatment in FHH, a randomized controlled trial is warranted.

## Consent

Written informed consent was obtained from the four patients for publication of this case series and any accompanying images. A copy of the written consent is available for review by the Editor-in-Chief of this journal.

## Competing interests

The authors declare that they have no competing interests.

## Authors' contributions

PS performed the medical examination and initiated the cinacalcet treatment regarding the FHH patients. AQR analyzed and interpreted the patient data and was a major contributor in writing the manuscript. NRJ contributed in writing the manuscript. All authors read and approved the final manuscript.

## References

[B1] Schwarz P Sorensen HA Transbol I Inter-relations between the calcium set-points of Parfitt and Brown in primary hyperparathyroidism: a sequential citrate and calcium clamp study Eur J Clin Invest 1994 24 553 558 10.1111/j.1365-2362.1994.tb01106.x 7982443

[B2] Brown EM Familial hypocalciuric hypercalcemia and other disorders with resistance to extracellular calcium Endocrinol Metab Clin North Am 2000 29 503 522 10.1016/S0889-8529(05)70148-1 11033758

[B3] Brown EM Clinical utility of calcimimetics targeting the extracellular calcium-sensing receptor (CaSR) Biochem Pharmacol 2010 80 297 307 10.1016/j.bcp.2010.04.002 20382129

[B4] White E McKenna J Cavanaugh A Breitwieser GE Pharmacochaperone-mediated rescue of calcium-sensing receptor loss-of-function mutants Mol Endocrinol 2009 23 1115 1123 10.1210/me.2009-0041 19389809 PMC2703600

[B5] Peacock M Bilezikian JP Bolognese MA Borofsky M Scumpia S Sterling LR Cheng S Shoback D Cinacalcet HCl reduces hypercalcemia in primary hyperparathyroidism across a wide spectrum of disease severity J Clin Endocrinol Metab 2011 96 E9 E18 10.1210/jc.2010-1221 20943783 PMC3203649

[B6] Shoback DM Bilezikian JP Turner SA McCary LC Guo MD Peacock M The calcimimetic cinacalcet normalizes serum calcium in subjects with primary hyperparathyroidism J Clin Endocrinol Metab 2003 88 5644 5649 10.1210/jc.2002-021597 14671147

[B7] Moe SM Cunningham J Bommer J Adler S Rosansky SJ Urena-Torres P Albizem MB Guo MD Zani VJ Goodman WG Sprague SM Long-term treatment of secondary hyperparathyroidism with the calcimimetic cinacalcet HCl Nephrol Dial Transplant 2005 20 2186 2193 10.1093/ndt/gfh966 16030053

[B8] Silverberg SJ Rubin MR Faiman C Peacock M Shoback DM Smallridge RC Schwanauer LE Olson KA Klassen P Bilezikian JP Cinacalcet hydrochloride reduces the serum calcium concentration in inoperable parathyroid carcinoma J Clin Endocrinol Metab 2007 92 3803 3808 10.1210/jc.2007-0585 17666472

[B9] Peacock M Bolognese MA Borofsky M Scumpia S Sterling LR Cheng S Shoback D Cinacalcet treatment of primary hyperparathyroidism: biochemical and bone densitometric outcomes in a five-year study J Clin Endocrinol Metab 2009 94 4860 4867 10.1210/jc.2009-1472 19837909

[B10] Lewiecki EM Management of skeletal health in patients with asymptomatic primary hyperparathyroidism J Clin Densitom 2010 13 324 334 10.1016/j.jocd.2010.06.004 21029971

[B11] Timmers HJ Karperien M Hamdy NA de BH Hermus AR Normalization of serum calcium by cinacalcet in a patient with hypercalcaemia due to a *de novo *inactivating mutation of the calcium-sensing receptor J Intern Med 2006 260 177 182 10.1111/j.1365-2796.2006.01684.x 16882283

[B12] Festen-Spanjer B Haring CM Koster JB Mudde AH Correction of hypercalcaemia by cinacalcet in familial hypocalciuric hypercalcaemia Clin Endocrinol (Oxf) 2008 68 324 325 10.1111/j.1365-2265.2007.03027.x 17803689

[B13] Alon US Vandevoorde RG Beneficial effect of cinacalcet in a child with familial hypocalciuric hypercalcemia Pediatr Nephrol 2010 25 1747 1750 10.1007/s00467-010-1547-5 20495831

[B14] Reh CM Hendy GN Cole DE Jeandron DD Neonatal hyperparathyroidism with a heterozygous calcium-sensing receptor (CASR) R185Q mutation: clinical benefit from cinacalcet J Clin Endocrinol Metab 2011 96 E707 12 10.1210/jc.2010-1306 21289269

[B15] Schwarz P Larsen NE Lonborg Friis IM Lillquist K Brown EM Gammeltoft S Familial hypocalciuric hypercalcemia and neonatal severe hyperparathyroidism associated with mutations in the human Ca2+-sensing receptor gene in three Danish families Scand J Clin Lab Invest 2000 60 221 227 10.1080/003655100750044875 10885494

